# Hepatitis C virus and host cell nuclear transport machinery: a clandestine affair

**DOI:** 10.3389/fmicb.2015.00619

**Published:** 2015-06-19

**Authors:** Barbara Bonamassa, Francesco Ciccarese, Veronica Di Antonio, Andrea Contarini, Giorgio Palù, Gualtiero Alvisi

**Affiliations:** ^1^Department of Molecular Medicine, University of Padua, Padua, Italy; ^2^Veneto Institute of Oncology IOV-IRCCS, Padua, Italy

**Keywords:** nucleoporin, importin, exportin, Ran, nuclear import, caspase, replication factories, NPC

## Abstract

There is growing evidence that factors encoded by cytoplasmic replicating viruses functionally interact with components of the nucleocytoplasmic transport apparatus. They do so either to access the cell nucleus, thus affecting genes expression, or to interfere with nuclear transport functionality, hindering host immune response. Recent studies revealed that the hepatitis C virus (HCV) makes no exception, interacting with the host cell nuclear transport machinery at two different levels. On the one hand, small amounts of both core and NS5A localize within the host cell nucleus during productive infection, modulating gene expression and signaling functions to promote persistent infection. On the other hand, HCV infection causes a profound redistribution of certain nucleoproteins to the close proximity of endoplasmic reticulum membrane-derived viral replication factories, where viral RNA amplification occurs. These nucleoporins are believed to form nuclear pore complex-like structures, as suggested by their ability to recruit nuclear localization sequence-bearing proteins. Thus, both processes are linked to virus-induced persistence and pathogenesis, representing possible targets for the development of novel anti-HCV therapeutics.

## Nuclear Transport

The eukaryotic nucleus is separated from the cytoplasm by a double membrane called nuclear envelope (NE). Nucleocytoplasmic transport occurs through NE-embedded nuclear pore complexes (NPCs). Cargoes >9 nm in diameter (∼60 kDa) are actively transported through NPCs by kariophilic transporters (Kaps) in a signal-mediated process. Such signals can be either nuclear localization or nuclear export sequences (NLSs and NESs), responsible for targeting into and out of the nucleus as mediated by importins (IMPs) or exportins (EXPs), respectively (Tran et al., 2007). Several types of NLSs exist, differing in their sequence and IMP-binding properties. Short, basic NLSs similar to that of the Simian Virus 40 Large Tumor Antigen (Tag) are referred to as “classical” NLSs (Kalderon et al., 1984). Such signals are recognized by IMPβ1 (IPO1) *via* one of the seven IMPα family members (IPOA1, IPOA3-8), which are classified in three subgroups according to their homology and cargo-binding specificity (Alvisi and Jans, 2015). Alternatively, IMPβ1 and its homolog can also bind cargoes directly without the need of IMPαs (Truant and Cullen, 1999; Fontes et al., 2000; Chook and Suel, 2011). These include IMPβ2 (IPO2), mainly responsible for importing RNA-binding proteins, and IMPβ3 (IPO5), involved in transport of core histones and ribosomal proteins (Jakel and Gorlich, 1998). All IMPβ homolog share similar mechanisms for cargo nuclear transport and release. Assembly of cargo-IMP complexes results in docking to the NPC, as mediated by IMPβ interaction with specific nucleoporins (Nups), followed by NPC translocation. Once inside the nucleus, binding of Ran-GTP to IMPβ mediates a conformational change, resulting in the dissociation of the complexes (Cansizoglu et al., 2007; Imasaki et al., 2007). Conversely, EXPs such as CRM-1 (XPO1) recognize NES-bearing cargoes in the nucleus upon binding to Ran-GTP, and translocate to the cytosol.

Nuclear replicating viruses interact with the nuclear transport apparatus to ensure nuclear targeting of viral replicating enzymes (Alvisi et al., 2011b, 2013). Strikingly, several proteins encoded by cytoplasmic-replicating viruses similarly interact with the nuclear transport apparatus, mainly with the aim to interfere with host cell functionality, promoting their self-propagation (Camus-Bouclainville et al., 2004; Zhang et al., 2007; Alvisi et al., 2008; Wang et al., 2012). Among these, the hepatitis C virus (HCV) makes no exception.

## Hepatitis C Virus

**FIGURE 1:**
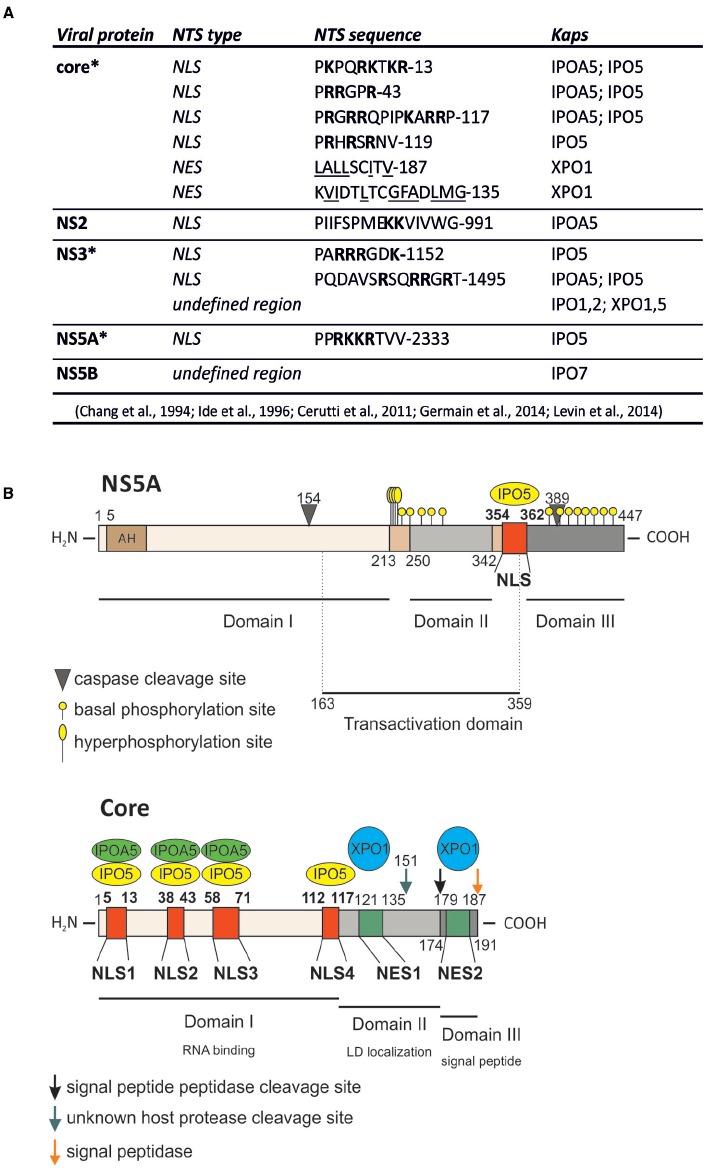
**HCV proteins contain functional nuclear transport signals (NTSs). (A)** A summary of HCV proteins binding to Kaps, as well as the sequences of identified NTSs. Basic residues within NLSs are in boldface; hydrophobic residues within NESs are underlined; the single letter amino acid code is used; *protein that can be detected within the host cell nucleus during viral infection. **(B)** Schematic representation of core and NS5A proteins, their functional domains as well as their NTSs and the Kap-binding sites.

Hepatitis C virus is a cytoplasmic-replicating RNA virus belonging to the *Flaviviridae* family. Once inside the host cell, its ∼9.6 Kbp genome is translated on the rough endoplasmic reticulum (ER) into a single polyprotein of about 3000 amino acids, co- and post-translationally cleaved by viral and host proteases into three structural proteins (core, E1, and E2), the viroporin p7, and six non-structural proteins (NS2, NS3, NS4A, NS4B, NS5A, and NS5B; Kato, 2000). Core, E1 and E2 constitute the viral particle, p7 and NS2 are involved in virion assembly and release, while NS3 to NS5B proteins are sufficient for RNA replication (Lohmann et al., 1999; Jones et al., 2007; Steinmann et al., 2007). Expression of the HCV polyprotein induces profound membrane rearrangements, including the formation of double membrane vesicles (DMVs) where viral proteins and host factors are recruited to replicate viral RNA (Romero-Brey et al., 2012). Such membrane alterations, i.e., viral replication factories (VFs), are common amongst positive-stranded RNA viruses, and are surrounded by autophagosomes and lipid droplets (LDs) in the case of HCV infection. Although their precise structure and function are still not completely clear, they support viral infection by a variety of mechanisms (Paul and Bartenschlager, 2013), such as by tethering the viral RNA during unwinding (Lyle et al., 2002) and by ensuring compartmentalization and concentration of viral products (Paul and Bartenschlager, 2013). VFs also provide lipids essential for replication (Ahola et al., 1999), and facilitate avoidance of host cell cytoplasmic pattern recognition receptors crucial for initiating antiviral immune responses (Overby et al., 2010). Furthermore, HCV-induced VFs have been recently shown to protect viral RNA from double strand RNA-induced host defenses and nucleases (Paul et al., 2013). Upon HCV infection, development of VFs is mainly dependent on NS4B and NS5A, the latter being responsible for DMV biogenesis *via* stimulation of the type III phosphatidylinositol 4-kinase α (PI4KIIIα; Reiss et al., 2011; Berger et al., 2014). Activated PI4KIIIα causes a profound redistribution of phosphatidylinositol-4 phosphate from the plasma membrane to VFs, where RNA polymerase NS5B-mediated HCV RNA replication occurs (Lohmann et al., 1999; Bianco et al., 2012). Newly synthesized viral genomes are subsequently delivered by NS5A on the surface of core-decorated LDs to be packaged in nucleocapsids, before being secreted through the very low density lipoprotein pathway (Miyanari et al., 2007; Gastaminza et al., 2008; Alvisi et al., 2011a). Although HCV life cycle has no clear nuclear intermediate, certain HCV proteins contain multiple functional NLSs and NESs, and interact with several Kaps (Figure 1; Chung et al., 2000; Germain et al., 2014; Levin et al., 2014). This redundancy of signals probably reflects the need for certain HCV proteins to be functionally targeted to different subcellular compartments according to specific stages of HCV life cycle. This would be consistent with the very recent discovery that HCV infection causes partial redistribution of certain Nups to the close proximity of VFs that are the sites of NLS-bearing cargo recruitment (Neufeldt et al., 2013; Levin et al., 2014).

## Functional Role of NS5A and Core Within the Cell Nucleus

**FIGURE 2:**
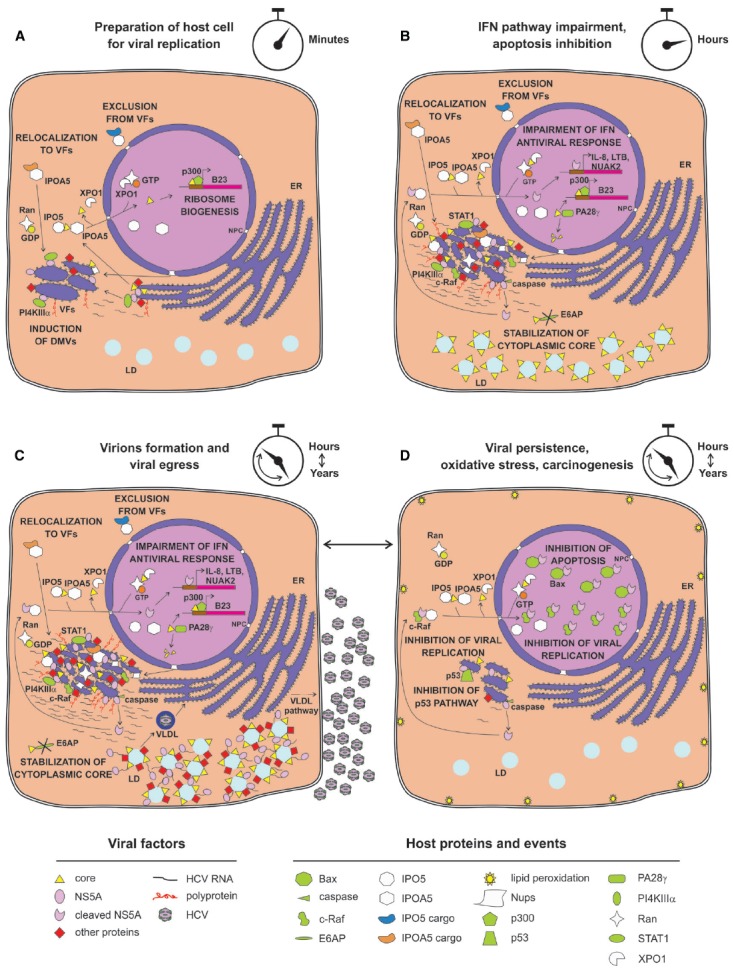
**Possible relationship between nuclear transport apparatus and HCV life cycle. (A)** Soon after HCV infection, core is transported into the nucleus to promote ribosome biogenesis. Newly synthesized viral proteins promote VF development, depending on NS5A-mediated recruitment of PI4KIIIα. Simultaneously, specific Nups are recruited by core and NS5A to VFs. **(B)** Small amounts of NS5A reach the nucleus after being processed by basal caspase activity, resulting in activation of a specific transcriptional program aimed at inhibiting IFN response and apoptosis. Core migrates to the nucleus to be degraded by PA28γ instead, thus stabilizing cytoplasmic core. **(C)** Viral replication progresses and specific host factors are recruited to VFs, either directly by viral proteins or indirectly by IMPs recognizing IPO5-dependent NLSs. **(D)** Strong viral replication and production eventually causes oxidative stress and lipid peroxidation that is detrimental to host cells, leading to activation of caspase activity and NS5A nuclear accumulation, thereby sequestering the host dependency factor c-Raf from VFs. This impairs viral replication and the pro-apoptotic factor Bax, thus promoting infected cells survival, viral reactivation, and liver damage.

So far, among all the HCV proteins bearing functional NLSs/NESs, insights into nuclear function has been delineated only for the NS5A and the core protein. NS5A is a multifunctional phosphoprotein directly involved in multiple stages of viral life cycle, including VFs biogenesis, viral RNA replication and particle assembly (Shimakami et al., 2004; Masaki et al., 2008; Reiss et al., 2011). Consequently, NS5A has been proposed to act as a master regulator of HCV life cycle, controlling the switch between viral replication and particle assembly through a still uncharacterized phosphorylation-dependent mechanism (Masaki et al., 2008). Additionally, NS5A affects a number of host cell signaling pathways, as exemplified by its ability to interact with several cellular proteins and regulate their activities (Pichlmair et al., 2012; Eberle et al., 2014). NS5A nuclear role seems to be primarily linked to HCV ability to establish a persistent infection, avoiding the host cell response and simultaneously enhancing the cellular survival ability (Figure 2). Indeed, nuclear NS5A impairs both interferon (IFN) antiviral and apoptotic pathways (Khabar et al., 1997; Ghosh et al., 1999; Majumder et al., 2001). NS5A bears a C-terminally-located NLS (Figure 1), which interacts with IPO5 and confers nuclear localization when fused to heterologous proteins (Ide et al., 1996; Chung et al., 2000; Levin et al., 2014). However, full-length NS5A is tethered to the ER by its N-terminal amphipathic α-helix (AH), which prevents nuclear translocation (Elazar et al., 2003). Importantly, NS5A contains two caspase cleavage sites at residue position 154 and 389 (Figure 1). In HCV-infected cells, basal caspase activity generates low levels of NS5A truncated forms in the absence of apoptotic stimuli. These include nuclear-accumulating fragments containing the NLS but lacking the AH, which bind the promoters of interleukin-8 (a negative regulator of the IFN pathway), as well as of the apoptosis inhibitors lymphotoxin beta and NUAK2, up-regulating their transcription (Satoh et al., 2000; Legembre et al., 2004; Haybaeck et al., 2009; Sauter et al., 2009; Maqbool et al., 2013). NS5A ability to migrate to the nucleus and modulate host gene expression appears to be important for HCV life cycle. Indeed, the degree of transcriptional activation mediated by different NS5A variants correlates with the levels of viral replication in the HCV replicon system. Furthermore, pharmacological ablation of caspase activity negatively affected HCV genome replication, preventing NS5A nuclear translocation (Maqbool et al., 2013). Besides transcriptional regulation, nuclear NS5A promotes cell survival also by preventing translocation of the apoptotic activator Bcl-2 associated X protein (Bax) to the nuclear membrane in response to cellular stress, thus mitigating its pro-apoptotic function (Chung et al., 2003; Ruggieri et al., 2012; Lindenboim et al., 2014). Furthermore, it has been recently shown that overexpression of NS5A N-terminally truncated forms impairs HCV replication, by relocalizing the host dependency factor c-Raf from VFs to cell nuclei (Sauter et al., 2009). Since c-Raf facilitates viral replication through attenuation of the IFN pathway, its withdrawal from VFs impairs HCV replication (Zhang et al., 2012). These findings probably reflect an auto-inhibitory mechanism evolved by HCV to reduce its own replication in case of virus-induced cellular stress, thus preventing apoptosis and promoting persistent infection. This is consistent with the ability of several HCV genotypes to decrease viral replication levels once viral-induced lipid peroxidation threatens cell viability (Yamane et al., 2014).

As mentioned above, important nuclear functions are also emerging for the core protein, a small structural protein forming the viral nucleocapsid and undergoing multiple maturation steps. Upon translation on the rough ER, core protein is initially cleaved from the nascent polyprotein by the host cell signal peptidase. This immature form of core is anchored to the ER-membrane by a C-terminal signal peptide, which facilitates translocation of the polyprotein into the ER lumen. Subsequently, it is cleaved by the intermembrane host cell signal peptide peptidase between residue 177 and 178 to enable the mature form of core to migrate on LDs (Ogino et al., 2004; Hope et al., 2006; Okamoto et al., 2008). Once there, core encapsidates newly generated viral genomes in concerted action with other viral proteins such as NS5A, NS2 and p7 (Appel et al., 2008; Stapleford and Lindenbach, 2011). In contrast to its predominantly cytoplasmic localization upon overexpression in cell culture or in productively infected cells *in vitro*, the core protein has been detected in the nucleus of hepatocytes of chronically infected patients and core transgenic mice (Moriya et al., 1997). Indeed, it contains four N-terminally located NLSs and two C-terminally located NESs (Figure 1). Such signals are functional, conferring both nuclear shuttling and Kap-binding abilities to reporter proteins (Cerutti et al., 2011; Levin et al., 2014). Importantly, immuno-electron microscopic analysis of HCV-infected hepatocytes allowed detection of small amounts of core into the cell nucleus 20 min post-infection. Additionally, core could also be observed in the nucleus later on, upon pharmacological inhibition of CRM-1, indicating that both NES and NLS are functional in the context of productively infected hepatocytes. Intriguingly, core could be detected in the nucleus in the absence of CRM-1 inhibition also upon knock-down of the proteasome activator PA28*γ*, which is responsible for ubiquitin-independent degradation of core in the cell nuclei. PA28*γ* inactivation also caused increase in the ubiquitination of cytoplasmic core, and impaired viral assembly (Moriishi et al., 2010; Cerutti et al., 2011). Indeed, core can be degraded both in the nucleus in an ubiquitin-independent manner, and in the cytosol through an ubiquitin-dependent process involving the E3 ubiquitin ligase E6AP (Suzuki et al., 2009; Shoji, 2012). Therefore, nuclear activation of PA28*γ* by core inhibits E6AP, resulting in increased overall core stability and promoting viral assembly and release (Moriishi et al., 2010). Additionally, core is endowed with transcriptional activity, and represents the only HCV protein globally regulating host cell transcription, by directly binding the TATA-binding protein (Moriishi et al., 2003; Kao et al., 2004). This transcriptional stimulation is further supported by the ability of core to promote ribosome biogenesis, supporting cell growth and viral replication through epigenetic enhancement of the nucleolar protein B23 transcription. Indeed, core can recruit the histone acetyltransferase p300 at the B23 promoter (Mai et al., 2006). In addition, nuclear core also inhibits transcription of genes linked to apoptosis, by up-regulating the expression of inhibitor of caspase-activated DNase (Sacco et al., 2003).

Therefore, both core and NS5A can localize in the cell nucleus and modulate transcription, promoting cell survival. In addition, NS5A also promotes immune evasion, while core specifically increases the translation rate of infected cells via increased ribosome biogenesis.

## Cytosolic Relocalization of Nups Upon HCV Infection

Hepatitis C virus VFs are a separate compartment from the cytoplasm (Miyanari et al., 2003; Hsu et al., 2010). Although the existence of a selective permeable barrier between the VF lumen and the surrounding cytoplasm has been postulated long ago, functional proof lacked until very recently (Neufeldt et al., 2013). Eventually, its definition has allowed reconciling the lack of HCV nuclear replication intermediate with the presence of functional NLSs and NESs within HCV proteins that are not observed in the nucleus during viral life cycle (Levin et al., 2014).

Various HCV proteins interact with high affinity nuclear transport factors (NTFs), although only core and NS5A functionally localize, to some extent, in the cell nuclei during viral infection (Moriya et al., 1997; Sauter et al., 2009; Cerutti et al., 2011; Maqbool et al., 2013; Germain et al., 2014; Levin et al., 2014). These findings suggested that HCV protein interaction with NTFs might be broader than originally thought. Mounting experimental evidence suggests that functional NPC-like structures could be recruited by HCV within the cytoplasmic VFs to build up a permeability barrier that improves the viral fitness (Neufeldt et al., 2013). While similar structures have been previously described within several cell types and are induced by other viruses, their functional characterization has begun only recently, using the HCV infectious system (Merisko, 1989; Marshall et al., 1996; Wang et al., 1997; Neufeldt et al., 2013). In particular, Nups representing most of the major NPC subcomplexes formed cytoplasmic foci upon HCV infection, and either co-localized or physically interacted with core and NS5A. Interestingly, this redistribution is associated with up-regulation at both the mRNA and protein level of specific Nups, further supporting the previously described HCV transcriptional regulation ability. Similarly, both IPOA5 and IPO5 partially relocalized to VFs upon HCV infection and interacted with several HCV proteins, although recruitment of Kaps to sites of HCV replication did not correlate with their over-expression, as in the case of Nups. Importantly, addition of NLS-mimicking peptides was sufficient to prevent the interaction of core and NS5A with IPO5/IPOA5 but not with Nups, suggesting that the interaction of viral proteins with Nups was NLS- and Kap-independent (Neufeldt et al., 2013). Knock-down of Nups and Kaps revealed that different NTFs play specific roles in HCV life cycle. Indeed, depletion of Nup98 and 153 strongly impaired viral replication, whereas down-regulation of Nup155 and IPO5 specifically affected viral particle assembly. As mentioned above, several HCV proteins have functional NLSs capable of interacting with different Kaps, including IMPα and IMPβ homolog such as IPOA5 and IPO5 (Germain et al., 2014; Levin et al., 2014). Intriguingly, addition of cell permeable HCV NLS-mimicking peptides binding to specific Kaps differently affected the outcome of viral infection. While IPOA5-binding peptides specifically impaired viral replication, IPO5-binding ones interfered with both viral replication and assembly (Levin et al., 2014). Importantly, functionality of the HCV-induced cytoplasmic Kap-Nup network was demonstrated by the delocalization of the small GTPase Ran to NS5A positive foci, and by the fact that such complexes were able to recruit a specific subset of cargoes (Neufeldt et al., 2013; Levin et al., 2014). Indeed, reporter proteins fused to IPOA5-binding NLSs (such as Tag NLS) were efficiently transported to core positive foci during productive infection, while IPO5-binding NLSs (such as Rev NLS) were not. This raises the possibility that IPOA5 and IPO5 may contribute to distinct processes during the HCV life cycle, respectively allowing and restricting trafficking of specific cargoes in and out of VFs (Levin et al., 2014).

Overall, these findings would support the redirection of Nups to cytoplasmic NPC-like structures and movement of viral and host NLS-bearing proteins from the cytoplasm to the lumen of VFs with the purpose to create isolated and protected niches of viral replication and assembly (Figure 2). The VF-associated NPCs could also be able to discriminate between NTFs, importing IPOA5-shuttled proteins in a selective way and excluding IPO5 cargos as well as NLS-lacking proteins from the HCV replication complexes. Therefore, it is possible that HCV exploits the nucleocytoplasmic transport system to target specific cargoes of viral and cellular origin to the nucleus and VFs. In this context, NS5A interaction with IPO5 would suggest that NPC-like structures play a role in triggering the switch between viral replication and assembly by restricting NS5A from entering the VFs. Since infection causes a decrease in the levels of nuclear accumulation of IPOA5-transported cargoes such as GFP-Tag NLS by partially relocalizing them to core positive foci, it is also possible that HCV-induced NPCs similarly impair nuclear targeting of specific host factors such as p53 or STAT1, thus interfering with their functions (Germain et al., 2014). Indeed, altered host cell nuclear transport and functions are not uncommon upon viral infections (Yarbrough et al., 2014).

It remains to be investigated how the two Kap-mediated transport systems affect each other in HCV-infected hepatocytes and whether trafficking to VFs differs from nuclear import at the molecular level. For sure, the striking news about HCV life cycle is that Nups do work outside the physiological environment of the NE during viral infection. The molecular basis responsible for functional and regulatory differences between the NPC-dependent transport systems located on VFs *or* NE are elusive at present (including the role of the GTPase Ran in VF import), which requires further experimental efforts.

## Conclusion

Detection of core- and NS5A-dependent transcriptional regulation and of cellular NTF-HCV protein interactions suggests that HCV exploits previously unidentified and nucleus-linked mechanisms to regulate both its survival and liver damage. These findings are consistent with the functionality of numerous NLSs found on HCV proteins. On the one hand, these NLSs are employed for protein shuttling to the nucleus. Consistently, core and NS5A and/or their variants regulate the cellular transcriptional environment, making it conductive to cell survival and prone to persistent viral infection by promoting ribosome biogenesis, and inhibiting IFN antiviral pathway and apoptotic cell death. On the other hand, NPC-like structures are proposed to form channels across the VF double membrane structures, which arise upon the HCV-induced cytoplasmic redistribution of Nups and Kaps. Selective trafficking across them regulates viral infection during distinct phases of HCV life cycle, with IPOA5- and IPO5-cargos being allowed or denied VF entry, respectively.

The further characterization of HCV trafficking signals has the potential to support the design of selective viral NLS/NES-targeted molecules with pangenotypic antiviral activity, targeting conserved regions among the HCV genotypes. Potentially, the same holds true for the discovery of new pieces of the cellular transcriptional puzzle altered by HCV, which may further help to forecast therapeutic outcomes after antiviral treatment.

### Conflict of Interest Statement

The authors declare that the research was conducted in the absence of any commercial or financial relationships that could be construed as a potential conflict of interest.
